# 1531. Early Real-World Experience of Long-Acting Cabotegravir (CAB) for HIV Pre-Exposure Prophylaxis (PrEP) in a Large Community-Based Clinic Network (CAN Community Health): Utilization and PrEP Persistence

**DOI:** 10.1093/ofid/ofad500.1366

**Published:** 2023-11-27

**Authors:** Jessica A Altamirano, Prerak Shukla, Steven K Barnett

**Affiliations:** CAN Community Health, Miami, Florida; CAN Community Health, Miami, Florida; CAN Community Health, Miami, Florida

## Abstract

**Background:**

Cabotegravir (CAB) is the first long-acting injectable form of HIV pre-exposure prophylaxis (PrEP) approved by the FDA on December 20, 2021. We assessed real-world utilization and adherence to Cabotegravir for PrEP in an outpatient community-based network in the United States (US).

**Methods:**

Electronic medical records were retrospectively reviewed from the CAN Community Health Network of 26 outpatient clinics across six US states to identify HIV-negative persons who received a prescription or injection for CAB for PrEP between December 2021 and April 2023. Demographics including age, race, ethnicity, sex assigned at birth, gender identity and sexual orientation were collected. Two groups were assessed: 1) those who received ≥ 1 Cabotegravir injection following a prescription and 2) those who were prescribed but never received any Cabotegravir injections. Rate of switching from oral PrEP to CAB injections, persistence and discontinuation of CAB injections were studied as well as HIV seroconversion. Discontinuation was defined as either a switch back to oral PrEP or being > 67 days from last CAB injection.

**Results:**

There were 293 HIV-negative individuals prescribed CAB for PrEP with median age of 36 years, 239 male and 54 female sex assigned at birth; 167 same-sex, 53 heterosexual, 48 bisexual; 97 White Non-Hispanics, 86 Black Non-Hispanics, 60 White Hispanics (Tables 1 & 2). There were 155 (52.9%) HIV-negative individuals who were prescribed CAB for PrEP with ≥ 1 injections received. Of these, 126 individuals (81.3%) demonstrated PrEP persistence and no seroconversions. Discontinuation rate was 29 (18.7%) (Table 2). Reasons for discontinuation and switch back to oral PrEP were insurance coverage gaps or cost of copay (7), side effects (6), conflicts with work schedule (2), not documented (1).

Table 1

**Figure:**
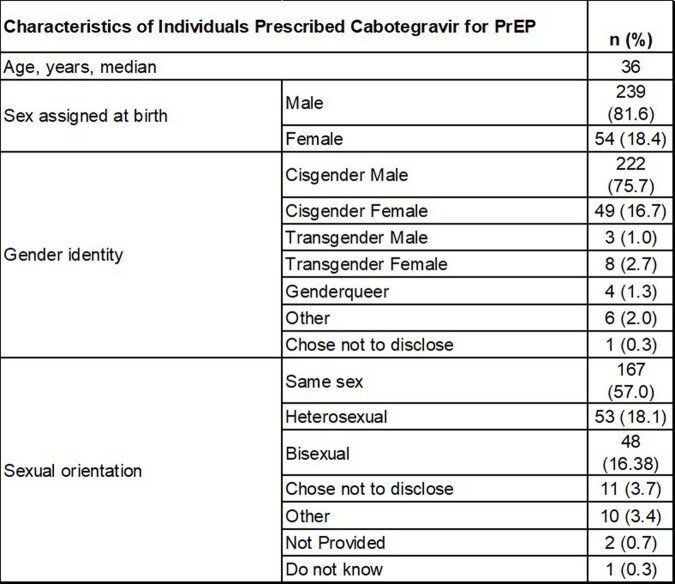

Table 2

**Figure:**
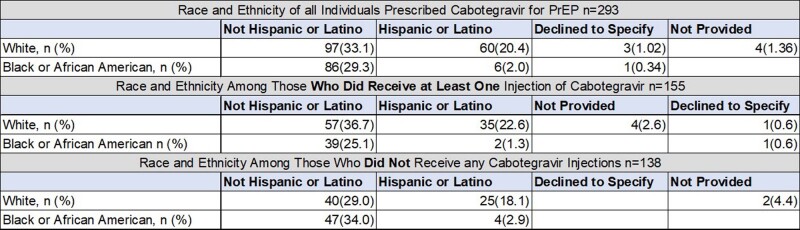

Table 3

**Figure:**
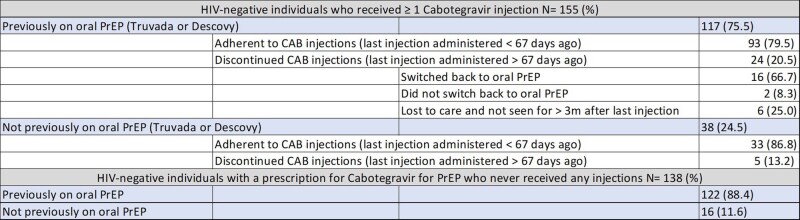

**Conclusion:**

Individuals prescribed Cabotegravir for PrEP reflected a diverse population. However, only 52.9% of prescriptions for Cabotegravir for PrEP resulted in at least one injection administered indicating limitations in use and access to injectable CAB for PrEP.

Those who did initiate CAB for PrEP showed a significant PrEP persistence percentage (81.3%) with an 18.7% rate of discontinuation attributed to insurance coverage gaps or side effects and less commonly conflicts with work schedule.

**Disclosures:**

**Jessica A. Altamirano, MD**, Janssen Pharmaceuticals: Speaker's Bureau

